# Mechanisms of Thromboinflammation in Viral Infections—A Narrative Review

**DOI:** 10.3390/v17091207

**Published:** 2025-09-03

**Authors:** Viviane Lima Batista, Jenniffer Ramos Martins, Celso Martins Queiroz-Junior, Eugenio Damaceno Hottz, Mauro Martins Teixeira, Vivian Vasconcelos Costa

**Affiliations:** 1 Department of Microbiology, Federal University of Minas Gerais, Belo Horizonte 30161-970, Brazil; vivianne.limab@gmail.com; 2 Department of Biochemistry and Immunology, Federal University of Minas Gerais, Belo Horizonte 30161-970, Brazil; jenniffermbio@gmail.com (J.R.M.); mmtexufmg@gmail.com (M.M.T.); 3 Department of Morphology, Federal University of Minas Gerais, Belo Horizonte 30161-970, Brazil; 4 Department of Immunothrombosis, Federal University of Juiz de Fora, Juiz de Fora 36036-900, Brazil; eugeniohottz@gmail.com

**Keywords:** thromboinflammation, homeostasis, viral infection, imbalance, thrombocytopenia

## Abstract

The circulatory and immune systems function in close coordination to maintain homeostasis and act as a frontline defense against infections. However, under certain conditions, this interaction becomes dysregulated, leading to thromboinflammation, a pathological process marked by the concurrent and excessive activation of coagulation, inflammation, and endothelial dysfunction. During viral infections, this phenomenon can markedly worsen clinical outcomes. Evidence indicates that viruses such as dengue, chikungunya, influenza, and SARS-CoV can trigger thromboinflammatory responses involving platelet activation, the release of procoagulant and pro-inflammatory mediators, and the formation of thrombi within blood vessels. While this response may initially help contain viral dissemination, in cases of high viremia it can progress to disseminated intravascular coagulation (DIC), hemorrhage, and multiple organ failure. This review compiles current evidence on thromboinflammatory mechanisms induced by arboviral and respiratory viruses and examines how these processes contribute to diseases’ pathogenesis and clinical severity.

## 1. From Homeostasis to Thromboinflammation: Mechanisms and Implications

The mammalian circulatory system performs essential biological functions, including oxygen delivery and defense against pathogens. It relies on a sophisticated hemostatic surveillance mechanism capable of rapidly sealing injuries to blood vessel walls, thereby preventing excessive blood loss and preserving the organism’s integrity [1,2]. Blood coagulation is a complex process in which platelets play a central role, working in concert with fibrin to form clots and prevent hemorrhage [3,4].

Platelets are small, anucleate, discoid cell fragments measuring approximately 2–3 μm in diameter, primarily responsible for regulating hemostasis. Beyond this role, they are essential in wound healing, angiogenesis, inflammation, innate immunity, and pathological thrombosis. They originate from the cytoplasm of megakaryocytes (MKs) located in the bone marrow [5,6], and the lungs [7]. Platelets play a critical role in maintaining vascular integrity, acting as sentinels for the detection of vessel injury. Upon vascular damage, they are rapidly recruited to the affected site through a tightly regulated process involving specific receptors’ interactions with components of the extracellular matrix [3,4,8,9]. Furthermore, during this process, activated platelets release the contents of their dense granules, including adenosine diphosphate (ADP) and serotonin, and their alpha granules, which contain inflammatory and coagulation factors, and express P-selectin on their surface, thereby amplifying the hemostatic response [3,4,8,9]. Activation of the coagulation cascade occurs in parallel with platelet activation. This process culminates in the formation of fibrin strands, a phenomenon known as secondary coagulation, which stabilizes the platelet plug. As extensively described by Engelmann et al. (2013) and Furie et al. (2008), this mechanism involves a series of reactions that lead to fibrin’s deposition and the amplification of the thromboinflammatory response [3,4].

The immune and hemostatic (coagulation) systems maintain a finely tuned balance, and their interaction is highly complex [4,10,11]. Under physiological conditions, this interplay is known as Immunothrombosis, in which immune cells and activated platelets, together with the coagulation system, act in concert to form blood clots. Conversely, when this response becomes dysregulated, it can progress to thromboinflammation, defined as the mutual amplification of thrombosis, inflammation, and vascular dysfunction [12]. This condition is a hallmark of several coagulopathies, disorders affecting blood coagulation that can lead to uncontrolled thrombosis and, in severe cases, multiple organ failure and death [10]. The concept of thromboinflammation is comprehensively reviewed by Waltraud C. et al. (2024) [11].

Thromboinflammation plays a crucial role in the pathogenesis of several diseases, including stroke [12], acute myocardial infarction [13], and preeclampsia [14]. In the context of viral infections, thromboinflammation is associated with the worsening of dengue [15], chikungunya [16], zika virus [17], yellow fever [18], influenza [19], and SARS-CoV [20]. This suggests that activation of the coagulation cascade during these infections has evolved in parallel with the immune system to help limit viral spread. However, acute viremia can trigger disseminated intravascular coagulation and/or hemorrhagic events, thereby worsening the clinical outcome and increasing mortality. In the following sections, we describe the thromboinflammatory mechanisms associated with each of these viruses.

## 2. Thromboinflammatory Mechanisms Induced by Arboviruses

Arboviruses comprise a diverse group of viruses transmitted by vectors, primarily arthropods such as mosquitoes. Among the most important vectors are *Aedes aegypti* and *Aedes albopictus*, which are responsible for the spread of several endemic diseases across multiple regions worldwide. Chikungunya and dengue are among the most prevalent and clinically significant arboviral diseases with systemic involvement [21].

### 2.1. Dengue

**Figure 1:**
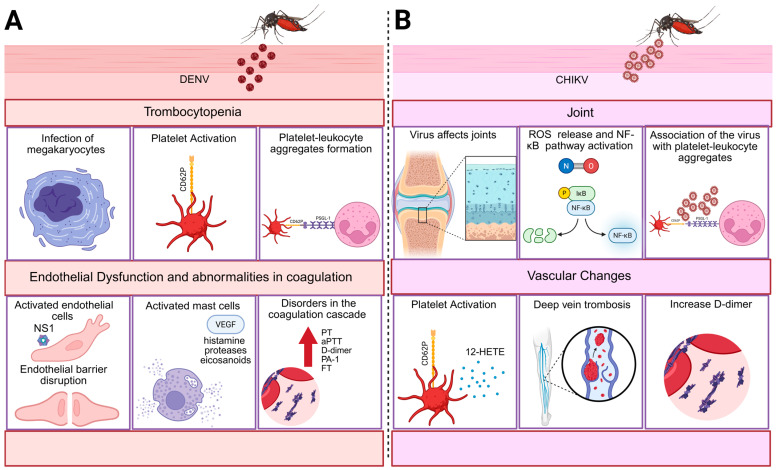
Thromboinflammatory mechanisms are induced by arboviruses. (**A**) Dengue. DENV triggers thromboinflammation through direct infection of bone marrow megakaryocytes, leading to cellular dysfunction and the release of immature, dysfunctional platelets. These platelets undergo premature activation and apoptosis. In peripheral blood, the virus can directly activate platelets and promote their interaction with leukocytes, enhancing clearance and contributing to thrombocytopenia. Both the virus and its NS1 protein also target endothelial cells, inducing activation, increased vascular permeability, and barrier disruption. In tissues, DENV activates resident mast cells, which release VEGF, histamines, proteases, and eicosanoids, amplifying inflammation and endothelial dysfunction. These events collectively disturb vascular homeostasis and coagulation. Clinically, severe dengue is characterized by prolonged PT and aPTT, elevated D-dimer levels (indicating fibrinolysis and risk of deep vein thrombosis), and increased expression of tissue factor and PAI-1, reflecting early coagulation cascade activation. (**B**) Chikungunya. The thromboinflammatory pathways triggered by CHIKV remain less defined. The virus exhibits tropism for joints, where chronic disease is linked to persistent synovial inflammation driven by nitric oxide production and NF-κB activation, leading to symptoms resembling rheumatoid arthritis. During the acute phase, CHIKV promotes platelet activation, P-selectin expression, and the formation of platelet–leukocyte aggregates, which may contribute to joint pain. Activated platelets also release 12-HETE, a pro-inflammatory lipid mediator also found in rheumatoid arthritis. These mechanisms may underline the elevated D-dimer levels observed in acute infection and the increased risk of thrombotic events, such as deep vein thrombosis, reported in some patients. Created in Biorender [31].

Dengue is an acute systemic viral infection caused by four genetically and antigenically related viruses, known as serotypes 1–4 (DENV-1 to DENV-4), each grouped into their respective genotypes [22]. These arboviruses belong to the genus *Orthoflavivirus*. Clinically, the disease is classified as dengue with or without warning signs, or as severe dengue (SD) [23]. Severe dengue is characterized by increased vascular permeability and a cytokine storm, followed by shock, which can be fatal [22,24,25,26]. Several severe manifestations of dengue are driven by hemostatic dysfunction and thromboinflammation through mechanisms involving (1) thrombocytopenia [27]; (2) formation of platelet–leukocyte aggregates [28]; (3) vascular and endothelial dysfunction [29]; and (4) coagulation abnormalities [30] (Figure 1 and Table 1).

#### 2.1.1. Dengue-Induced Thrombocytopenia

Thrombocytopenia is a common manifestation in dengue patients, occurring in both mild and severe cases, although its underlying mechanisms are not yet fully understood. Evidence suggests that it may result from decreased platelet production in the bone marrow and/or enhanced destruction and clearance in the peripheral blood [32]. Additionally, recent studies have shown that the lung is also a site of platelet biogenesis, and pulmonary injury may contribute to the development of thrombocytopenia [7,33]. In this context, dengue virus has been detected in the lung tissue of patients with severe disease, as revealed by post-mortem biopsy analyses [34]. Furthermore, several studies have demonstrated a positive association between platelet activation and thrombocytopenia during dengue infection [27,35,36,37].

Dengue virus (DENV) may impair platelet formation by interfering with megakaryopoiesis [38,39,40,41,42]. This occurs through direct infection of bone marrow megakaryocytes, activating the Notch signaling pathway, which is associated with suppression of megakaryopoiesis in human cells [43,44]. Moreover, DENV-infected megakaryocytes reprogram their transcriptome, modulating the expression of genes transferred to platelets during infection, including interferon-stimulated genes (ISGs), potentially affecting both antiviral responses [45] and thrombopoiesis [46,47]. Platelets derived from DENV-infected megakaryocytes may exhibit impaired aggregation, as indicated by a reduced expression of surface markers such as CD41, CD42a, and CD61. The downregulation of these markers is linked to defective platelet adhesion and contributes to the thrombocytopenia observed in dengue infection [39]. Another key aspect is that megakaryocytes undergo an endomitotic cell cycle, characterized by an S phase followed by aborted mitotic entry, during which anaphase and cytokinesis are entirely bypassed, resulting in irregular chromosomal segregation [38,48]. Evidence suggests that DENV suppresses megakaryocyte polyploidization through a mechanism that is not yet fully understood, impairing proper platelet formation and contributing to the thrombocytopenia characteristic of the infection [38].

Several studies have demonstrated that platelets harboring high numbers of DENV genome copies become hyperactivated, as evidenced by elevated P-selectin expression and increased PAC-1 antibody binding on their surface. These platelets also display enhanced deposition of complement C3 and IgG on their membrane factors, linked to platelet lysis and clearance [27,36,49]. DENV-2-activated platelets markedly promote clot formation, while phagocytic cells, including monocytes, macrophages, dendritic cells, and neutrophils, rapidly engulf these platelets in an activation-dependent manner, thereby contributing to the thrombocytopenia observed in dengue [27,50,51]. In addition, these platelets exhibit mitochondrial dysfunction and classical apoptotic features, including mitochondrial depolarization, activation of caspases-9 and -3, and increased phosphatidylserine exposure, the primary “eat me” signal recognized by macrophages [52]. Evidence also indicates that platelet clearance in the spleen is a key event in DENV-induced thrombocytopenia. Furthermore, serotonin released by mast cells during infection has been shown to play a pivotal role in platelet activation, thereby exacerbating hemostatic dysfunction [53]. Additional mechanisms contributing to dengue-associated thrombocytopenia have been comprehensively reviewed by Quirino-Teixeira (2022) and Khazali et al. (2024) [32,54].

Collectively, these findings indicate that DENV-induced thrombocytopenia results from a multifactorial process involving multiple organs and physiological pathways, including modulation of transcription factors and disruption of key stages in platelet production.

#### 2.1.2. Platelet–Leukocyte Aggregate Formation Mediated by P-Selectin

Platelet–leukocyte aggregates play a pivotal role in the pathogenesis of thromboinflammation and in the vascular complications observed in severe dengue cases [52]. Activated platelets amplify the inflammatory cascade by releasing pro-inflammatory and procoagulant mediators, including neutrophil-activating peptide 2 (NAP-2) and platelet-activating factor (PAF), thereby contributing to the exacerbation of inflammation and the thrombotic events associated with the disease [55]. Furthermore, both platelets and endothelial cells translocate P-selectin to their surface upon activation. This vascular adhesion molecule mediates interactions between activated platelets, endothelial cells, and leukocytes expressing P-selectin glycoprotein ligand-1 (PSGL-1), its primary receptor in vivo [56,57]. Engagement of P-selectin with PSGL-1 triggers the activation of β2 integrins on leukocytes, which in turn promotes their interaction with multiple ligands, such as platelet GPIbα, ICAM-2, and fibrinogen in the presence of thrombin, ultimately leading to full integrin activation [58].

Clinical evidence indicates that the formation of platelet–leukocyte aggregates, particularly platelet–monocyte aggregates (PMAs), is a frequent and dynamic event in dengue virus-infected patients, occurring in a biphasic pattern between days 6–8 and 11–16 after fever’s onset [59]. One study reported a significant increase in PMAs in dengue patients compared to healthy controls, with a positive correlation between platelet P-selectin expression and aggregate frequency [52]. PMAs were particularly pronounced in patients with thrombocytopenia and clinical signs of vascular leakage, such as hemoconcentration, hypoalbuminemia, and postural hypotension. Furthermore, PMA frequency negatively correlated with both platelet counts and serum albumin levels. Functionally, monocytes exposed to platelets from dengue patients secreted a range of pro- and anti-inflammatory cytokines, including IL-1β, IL-8, IL-10, and MCP-1, whereas platelets from healthy donors induced only MCP-1 production. These findings indicate that PMA formation contributes to the exacerbated inflammatory response and plays a key role in the pathophysiology of severe dengue [52]. The platelet–monocyte interaction is recognized as a key mechanism linking thrombosis and inflammation. Accordingly, PMAs are elevated in various thromboinflammatory diseases and correlate with disease severity [60,61,62].

In addition to monocytes, neutrophils act as key sentinel cells during dengue virus (DENV) infection. Evidence shows that DENV can activate neutrophils through serotype-independent mechanisms involving the C-type lectin receptor CLEC5A and Toll-like receptor 2 (TLR2), leading to NLRP3 inflammasome’s activation and subsequent release of neutrophil extracellular traps (NETs) and inflammatory cytokines [63,64,65]. Furthermore, DENV activates platelets via the CLEC2 receptor, promoting their aggregation with neutrophils and the release of extracellular vesicles that enhance NET formation and inflammatory mediator production through CLEC5A and TLR2 signaling in neutrophils and macrophages. Simultaneous blockade of CLEC5A and TLR2 inhibits NET formation, reduces inflammation, and markedly decreases DENV-induced systemic vascular permeability. These findings highlight the critical roles of the CLEC2 and CLEC5A/TLR2 pathways in platelet–leukocyte interactions and support the development of therapeutic strategies targeting immune modulation and tissue protection in severe dengue [64].

A recent study by Böer et al. (2024) evaluated the monocyte-to-lymphocyte ratio (MLR), neutrophil-to-lymphocyte ratio (NLR), and platelet-to-lymphocyte ratio (PLR) as potential prognostic inflammatory biomarkers in dengue patients. In a retrospective cohort of 193 patients treated in Brazil between 2012 and 2013, cases of SD were significantly associated with elevated MLR and reduced PLR, suggesting that an increased MLR combined with a decreased PLR may indicate a higher risk of progression to severe disease [66]. Additionally, platelets can interact with various lymphocyte populations, including T cells, B cells, and NK cells, via P-selectin- and integrin-mediated binding [67]. However, the role of platelet–lymphocyte aggregates in dengue’s pathogenesis remains incompletely understood. The dynamics of platelet–leukocyte aggregates have been further reviewed by Amin and colleagues (2025) [68] (Table 1).

P-selectin acts as a central mediator of thromboinflammation. In a sickle cell anemia model, blockade of P-selectin using an anti-CD62P monoclonal antibody reduced platelet–neutrophil aggregates and attenuated lung injury in mice [69]. Furthermore, studies in P-selectin-deficient (P-/-) mice have demonstrated that the absence of this molecule decreases formation of platelet–leukocyte aggregate and, consequently, development of thrombus in models of deep vein thrombosis [70,71]. Collectively, these findings highlight the critical role of P-selectin in leukocyte aggregation and thrombus formation.

#### 2.1.3. Endothelial Dysfunction

Severe dengue is characterized by a transient increase in vascular permeability due to endothelial dysfunction, predominantly occurring during the critical phase, which typically lasts 24–48 h [72,73]. Disruption of the endothelial barrier leads to excessive fluid leakage, a phenomenon known as hyperpermeability, clinically manifested as vascular leakage and fluid’s accumulation in surrounding tissues. As a result, arterial and pulse pressures decrease, compromising tissue perfusion and potentially causing organ dysfunction [73]. The pathogenesis of endothelial dysfunction in dengue is multifactorial, arising from complex interactions between viral factors and the host immune response. Among viral components, non-structural protein 1 (NS1), secreted during DENV infection, plays a central role. NS1 alone can induce endothelial hyperpermeability in vitro and vascular leakage in vivo, independently of viral infection. Moreover, NS1 degrades the endothelial glycocalyx-like layer (EGL) in vitro via activation of endothelial sialidases and the cathepsin L/heparanase pathway [74]. Additionally, NS1 enhances vascular permeability through Toll-like receptor 4 (TLR4) activation, leading to inflammatory cytokine production [75], and it stimulates endothelial cells to secrete macrophage migration inhibitory factor (MIF), which increases permeability via autophagy-mediated mechanisms [76]. Multiple cell types respond to NS1 by producing pro-inflammatory mediators, including TNF, IL-1β, matrix metalloproteinases (MMPs), vascular endothelial growth factor (VEGF), and platelets themselves, which collectively disrupt the endothelial barrier and enhance vascular permeability during DENV infection [75,77]. Moreover, various cell types, including platelets, respond to NS1 stimulation by producing pro-inflammatory mediators such as TNF, IL-1β, matrix metalloproteinases (MMPs), and vascular endothelial growth factor (VEGF) [78,79]. These molecules collectively contribute to endothelial barrier disruption and the increased vascular permeability observed during DENV infection [77,80,81]. Additionally, direct DENV infection of endothelial cells modulates surface adhesion molecules, including ICAM-1, VCAM-1, and E-selectin, facilitating leukocyte recruitment to the activated endothelium [82,83] (Table 1).

Mast cells play a central role in the pathogenesis of severe dengue, primarily through direct activation by DENV, which triggers the release of newly synthesized mediators such as histamine, mast cell-specific proteases, eicosanoids (including leukotrienes and prostaglandins), cytokines, and chemokines. These mediators act directly on the vascular endothelium, compromising its integrity and function [84]. In vitro studies have shown that human mast cells can be permissive to DENV infection in an antibody-dependent manner via the FcγRII receptor [85,86]. Dengue patients exhibit elevated histamine levels in plasma and urine, as well as increased concentrations of VEGF and mast cell-derived proteases [84,87,88]. The release of these granule-stored mediators promotes endothelial activation, and mast cell-derived chymase has been shown to contribute to vascular leakage in mouse models of DENV infection [89]. DENV-induced mast cell activation also enhances the production of chemokines such as CCL3, CCL4, and CCL5 [90], along with pro-inflammatory cytokines including IL-6, IL-1β, and TNF [89,91]. Importantly, TNF released by infected mast cells acts as a potent activator of endothelial cells, playing a central role in the pathogenesis of SD [86,91,92].

#### 2.1.4. Abnormalities in Coagulation

DENV infection is frequently associated with hemostatic disturbances, primarily driven by the host immune response. This response triggers simultaneous activation of coagulation and fibrinolysis pathways, leading to a thromboinflammatory state and multifactorial coagulopathy, particularly in severe cases [30,93,94]. Patients with severe dengue often present with thrombocytopenia, a prolonged prothrombin time (PT) and activated partial thromboplastin time (aPTT), and elevated plasma D-dimer levels, reflecting systemic hemostatic activation [95,96].

During convalescence, gradual normalization of hemostatic parameters reflects the restoration of hemostatic balance, with the degree of dysfunction correlating directly with disease severity [97]. Wills et al. (2002) reported marked reductions in anticoagulant proteins C, S, and antithrombin III in children with dengue, likely resulting from vascular leakage. Elevated levels of tissue factor (TF), thrombomodulin, and PAI-1 further indicate activation of the endothelium, platelets, and monocytes [37]. Systemic inflammation contributes to coagulopathy, as evidenced by Avila-Aguero et al. (2004), who found that fever, spontaneous bleeding, and a positive tourniquet test were associated with increased IL-6, IL-8, TNF, and coagulation activation [95]. In vitro, IL-6 increases endothelial permeability via MEK/ERK signaling [98], and animal models link elevations in IL-6 and IL-8 to increased D-dimer levels and thrombotic risk [99,100]. Elevated D-dimer reflects excessive coagulation followed by fibrinolysis and is associated with endothelial dysfunction, shock severity, and mortality in children with dengue shock syndrome [101]. Recently, Das et al. (2024) demonstrated that DENV NS1 downregulates coagulation factors I, V, X, and XIII through ERK-mediated transcriptional repression of HNF4α in Huh7 cells overexpressing NS1 [102]. The role of TF in hemorrhagic fevers has also been highlighted in Ebola virus infection: Geisbert et al. (2003) reported increased TF expression in monocytes and macrophages of infected primates, and its inhibition reduced lethality [103,104]. Normally absent from circulation, TF is a potent initiator of coagulation and represents a critical interface between inflammation and hemostasis [105,106].

In dengue, increased TF expression in monocytes from patients with severe disease has been reported, contributing to coagulopathy and thrombocytopenia [37,106]. Additionally, Huerta-Zepeda et al. (2008) observed upregulation of TF and protease-activated receptor-1 (PAR-1) in DENV-activated endothelial cells [107]. These findings suggest that coagulopathy in DENV infection results from a combination of immune activation, systemic inflammation, and endothelial dysfunction. Understanding the underlying molecular mechanisms, including the roles of NS1 and TF, may facilitate the development of targeted therapies to prevent the hemorrhagic and thrombotic complications associated with severe dengue.

#### 2.1.5. Thromboinflammation in Dengue: Pregnancy Context

Although most cases of SD are characterized by the thromboinflammatory mechanisms previously described, certain groups develop less common yet clinically significant complications. Pregnant women represent a particularly vulnerable population due to physiological and immunological changes during gestation that impair their ability to mount effective immune responses [108]. Furthermore, hormonal adaptations, such as progesterone-induced increases in body temperature and estrogen-mediated water retention, may enhance attractiveness to mosquitoes, thereby facilitating DENV transmission [108,109].

In this population, thromboinflammation plays a critical role, increasing the risk of adverse outcomes for both mother and fetus [110]. Although rare, vertical transmission of DENV, most frequently reported in endemic areas and associated with high viremia, occurs predominantly during the peripartum period [111,112,113]. Maternal thromboinflammatory manifestations, including thrombocytopenia, plasma leakage, and bleeding tendency, can compromise placental circulation, resulting in restrictions in fetal growth, low birth weight, or stillbirth. Increased vascular permeability during infection facilitates viral passage across the placental barrier, while capillary leakage syndrome induces ischemia, further impairing fetal development [113,114,115,116,117].

Studies suggest that placental hypoxia may induce trophoblastic epithelial damage, chorangiosis, and inflammatory changes in the decidua or chorion–decidua, thereby negatively impacting fetal growth [118,119]. Newborns from mothers with acute DENV infection may present with conditions ranging from mild febrile illness accompanied by thrombocytopenia to severe outcomes such as intracranial hemorrhage and neonatal death [120]. Systematic reviews and meta-analyses confirm that maternal dengue increases the risk of spontaneous abortion, preterm birth, and low birth weight [118,121].

## 3. Chikungunya

Chikungunya virus (CHIKV) is an Alphavirus belonging to the *Togaviridae* family, transmitted by *Aedes aegypti* and *Aedes albopictus* mosquitoes, which are also vectors for DENV [122,123]. CHIKV infection can be asymptomatic or symptomatic, with the latter commonly presenting with high fever, headache, fatigue, nausea, rash, and, most notably, intense arthralgia, a hallmark feature of the disease [122]. The chronic form of the disease, first described in 1979 and subsequently reported in later outbreaks, is defined by persistent or recurrent joint pain lasting weeks or months after the acute phase [124]. CHIKV exhibits tropism for joints and muscle tissues, triggering an inflammatory response in synovial joints, often leading to disabling pain and edema [125,126,127,128]. Chronic arthralgia usually affects the same areas involved in the acute phase and may mimic rheumatoid arthritis [124,128].

The immune and thromboinflammatory mechanisms driving persistent inflammation and musculoskeletal symptoms in CHIKV infection remain poorly understood. Recent evidence indicates that endothelial cells play a central role in vascular alterations in CHIKV-infected mice, where infection induces production of nitric oxide (NO) and reactive oxygen species (ROS) and activates the NF-κB pathway, suggesting that CHIKV complications may extend beyond the joints [16]. ROS are highly reactive oxygen-derived molecules, including superoxide anion, hydroxyl radical, and hydrogen peroxide [129]. At physiological levels, ROS contribute to redox signaling, cell proliferation, differentiation, and angiogenesis. However, excessive ROS production overwhelms cellular antioxidant defenses, resulting in oxidative stress and cell death [130].

Previous evidence also suggests that platelets may contribute to musculoskeletal symptoms. Electron microscopy images showed that CHIKV directly associates with human platelets and can be trapped within platelet aggregates. During this interaction, some platelets displayed degranulation and lysis [131,132]. Similarly, rabbit platelets exposed to CHIKV for 24 h showed significant degranulation and lysis, suggesting that CHIKV may induce platelet activation and cell damage in multiple models [131,132]. Additionally, increased expression of platelet P-selectin and elevated levels of soluble P-selectin have been observed in patients with Chikungunya [133]. The same study reported elevated plasma levels of 12-HETE, a metabolite of arachidonic acid. 12-HETE is known to modulate vascular and joint inflammation in both sterile and infectious diseases. In rheumatoid arthritis, increased neutrophil infiltration, platelet activation, and 12-HETE synthesis have been documented [134]. Given that rheumatoid arthritis-like symptoms may occur in chronic CHIKV infection, 12-HETE’s elevation could be linked to platelet activation. Supporting this, Quintanilha et al. (2022) showed that patients who developed chronic joint and muscle pain had higher platelet activation in the acute phase [133].

Vascular complications have also been reported in CHIKV infection [135]. A case report described deep vein thrombosis in a CHIKV-infected patient [136]. Another study assessed D-dimer levels in 141 PCR-confirmed CHIKV patients and found that 63.8% had elevated D-dimer levels, suggesting an association with activation of coagulation [17]. D-dimers are fibrin degradation fragments generated during fibrinolysis, and their elevation serves as a biomarker of clot formation and breakdown in thrombotic events [137]. Despite these findings, thromboinflammatory events during CHIKV infection remain underexplored. Given the established role of platelets in vascular pathology in other viral diseases, further investigation is needed to better understand their contribution to the pathogenesis of Chikungunya virus infection (Table 1).

## 4. Influenza

The influenza virus belongs to the *Orthomyxoviridae* family and is a single-stranded RNA virus with zoonotic potential, responsible for epidemics that cause approximately 5 million severe respiratory infections in humans each year [138]. It is primarily transmitted through respiratory droplets and contact with contaminated surfaces. Clinical manifestations range from asymptomatic or mild infections to severe forms that may progress to viral pneumonia, respiratory failure, and death, particularly in high-risk groups such as the elderly or individuals with comorbidities [139]. Influenza is the causative agent of flu and is classified into four types: A (associated with global pandemics), B, C, and D [140]. Influenza A virus (IAV) is of major global importance due to its high genetic variability and remarkable adaptability. It can infect not only humans but also a wide variety of animal species, including birds, pigs, horses, bats, and others [141]. IAV is classified based on its surface glycoproteins, hemagglutinin (HA) and neuraminidase (NA), resulting in subtypes such as H1N1, H3N2, H5N1, and H7N9. Among these, H1N1 and H3N2 are the most prevalent and widely disseminated among humans [142,143].

The virus enters host cells via endocytosis after its hemagglutinin binds to sialic acid on the surface of epithelial cells in the respiratory tract [144] and is recognized by Toll-like receptors (TLRs), which activate key immune cells such as macrophages, neutrophils, and dendritic cells [145]. Furthermore, the virus can be detected in circulation, where it is internalized by platelets via TLR7, leading to the release of complement C3. This, in turn, stimulates neutrophils to release their DNA, resulting in a highly prothrombotic process and increasing the risk of cardiovascular events. In parallel, platelets release GM-CSF, which favors neutrophil survival and the formation of platelet–neutrophil clusters. Consequently, these platelets become dysfunctional or reduced in number, impairing their ability to contain inflammation and further favoring viral spread [146]. This recognition leads to the production of pro-inflammatory cytokines, including TNF, IL-6, IL-8, and IFN-α/β, resulting in symptoms such as fever, cough, rhinorrhea, sore throat, myalgia and, in severe cases, acute respiratory distress syndrome (ARDS) and pulmonary failure [147]. This intense inflammatory response can cause tissue damage, particularly to the pulmonary endothelium, making it more susceptible to dysfunction. The pulmonary endothelium actively regulates key processes involved in lung homeostasis, such as angiogenesis, vasomotor tone, regulation of vascular permeability to macromolecules and leukocytes, blood coagulation, and the production of cytokines and adhesion molecules. These functions, however, may be impaired during ARDS [148].

Endothelial dysfunction during influenza infection is a complex event involving multiple mechanisms, including the following: (I) Increased expression of adhesion molecules associated with endothelial activation (e.g., VCAM-1 and ICAM-1), which contributes to enhanced leukocyte and platelet adhesion to the endothelium. These molecules bind to very late antigen-4 (VLA-4) and CD11/CD18 integrins, thereby facilitating leukocytes’ migration and adhesion to the endothelial surface [149]. (II) Reduced production of NO, a key molecule generated by nitric oxide synthase (NOS) activity. NO exerts inhibitory effects on platelet activation via signaling pathways, and its decreased availability is directly associated with enhanced platelet activation and the promotion of a procoagulant state [150]. (III) Excessive upregulation of TF, a protein involved in initiating the coagulation cascade following vascular injury. Aberrant expression of TF triggers a robust activation of the coagulation cascade, leading to thrombus formation, as previously reported in murine infections with IAV/H1N1 [151]. (IV) Exposure of extracellular matrix proteins (e.g., collagen). Under normal conditions, extracellular matrix proteins are found beneath the vascular endothelium; however, endothelial injury leads to their exposure to circulating blood, promoting interactions with platelets that facilitate their adhesion and activation. This process, in turn, amplifies both the inflammatory response and the coagulation cascade, as demonstrated in vitro experiments using human platelets [152].

Platelets play a crucial role in immunological homeostasis through both direct and indirect interactions with various immune cell types, such as leukocytes. However, their role during viral infections, particularly in the context of IAV, remains incompletely understood. Several case studies have demonstrated a correlation between influenza virus infection and thrombocytopenia. For example, children with severe influenza A and B infections exhibited a significantly reduced platelet distribution width (PDW) compared to those with non-severe disease [153]. Moreover, PDW has been associated with disease severity in patients with IAV-induced ARDS [148]. Platelets can engulf the virus via binding to sialoglycans, followed by neuraminidase-mediated removal of sialic acids, which facilitates hepatic clearance of platelets. This mechanism has been observed through the internalization of IAV in vitro. In vivo experiments using IAV/H1N1-infected animal models [154] indicated a severe disease phenotype in which viral load was inversely correlated with platelet count during the acute phase, highlighting an intrinsic role of IAV in the development of thrombocytopenia. A recent study demonstrated that to circumvent the deleterious effects of thrombocytopenia, hematopoietic stem cells (HSCs) can detect inflammatory signals and, independently of thrombopoietin, increase megakaryocyte production to restore platelet levels [155]. A potential mechanism of thrombocytopenia in experimental IAV infection is pulmonary microvascular thrombosis [156]. In this model, activation or inhibition of protease-activated receptor 4 (PAR4), a thrombin receptor, exacerbated or recovered, respectively, lung injury and mortality, which was also mitigated by genetic deletion or pharmacological inhibition of integrin αIIb/β3. Integrin αIIb/β3 is a surface receptor found on platelets and is responsible for fibrinogen-mediated platelet adhesion and aggregation, facilitating the formation of platelet aggregates and thrombi. In a study, PAC-1 (an antibody that binds to the active conformation of αIIbβ3 integrin) was used to assess platelet activation during H1N1 infection, and a significant increase was observed in infected patients, consistent with platelets in a hyperactivated state, inducing an inflammatory response (IL-6 production) and leukocyte/endothelial activation and increasing the risk of thrombosis and organ failure [157]. Thrombin was also directly involved in platelet activation in in vitro assays in which human platelets were stimulated with H1N1 virus in the presence of IAV immune serum [158]. Thrombin is a key enzyme in the coagulation cascade; it cleaves fibrinogen into fibrin, leading to clot formation.

**Figure 2:**
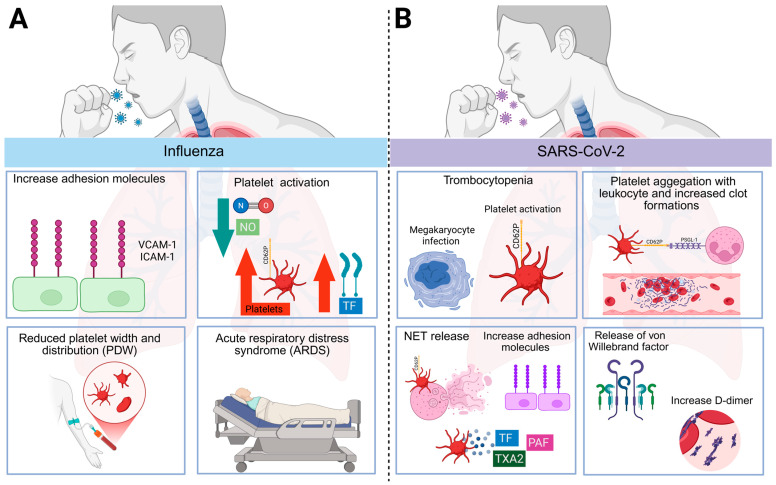
Respiratory virus-induced thromboinflammation. (**A**) Influenza virus. Influenza promotes endothelial dysfunction by upregulating adhesion molecules (VCAM-1, ICAM-1) and reducing nitric oxide (NO) production, a process linked to platelet activation and a procoagulant state. The infection also induces tissue factor (TF) expression, enhancing thrombus formation. Additionally, influenza alters platelet morphology by reducing platelets’ volume and size distribution, changes that correlate with disease severity and acute respiratory distress syndrome (ARDS). (**B**) SARS-CoV-2. SARS-CoV-2 can infect megakaryocytes and has been detected in the bone marrow of animal models, suggesting a mechanism for infection-associated thrombocytopenia. The virus also drives platelet activation and the formation of platelet–leukocyte aggregates, particularly with neutrophils, promoting neutrophil extracellular trap (NET) release and thrombosis. Elevated von Willebrand factor and D-dimer levels further indicate a hypercoagulable state. These events converge to cause endothelial dysfunction, characterized by increased adhesion molecule expression, and sustain the systemic thromboinflammatory response observed in COVID-19. Created in Biorender [164].

Although respiratory viruses such as IAV are known to impair lung function and can cause thrombocytopenia, the deleterious effects of infection on platelet precursor cells are still poorly understood. Megakaryocytes, platelet precursors, were recently found to be localized in the lungs, where they play an active role in thrombopoiesis [159]. IAV infection can lead to the development of ARDS, which is associated with a robust activation of the inflammatory response and the release of pro-inflammatory mediators [160]. Together, these events disrupt endothelial homeostasis, promoting platelet and leukocyte adhesion, platelet activation, activation of coagulation pathways, and clot formation, thereby exacerbating the thrombotic and inflammatory response [156]. These mechanisms may result in venous or arterial thrombosis [161,162], potentially leading to severe complications such as pulmonary embolism, stroke, or myocardial infarction. Although seasonal vaccines are available for different viral strains [163], severe infections still occur due to both viral and host-related factors. Understanding the underlying mechanisms is crucial for developing effective therapeutic strategies to manage thromboinflammatory complications associated with severe IAV infection and other respiratory diseases (Figure 2).

## 5. SARS-CoV-2

Coronaviruses (CoVs) belong to the *Coronaviridae* family, *Orthocoronavirinae* subfamily, and *Betacoronavirus* genus. They are associated with the development of severe acute respiratory syndrome (SARS) in infected individuals who progress to the most severe forms of the disease, characterized by atypical pneumonia, acute lung injury, fibrosis, and death [165]. Due to their high transmissibility, an epidemic caused by SARS-CoV-1 emerged between 2002 and 2003 in Guangdong Province, China, resulting in at least 770 deaths [166]. In late 2019, the appearance of SARS-CoV-2 in Wuhan, China, led to a global pandemic with immeasurable social and economic consequences. The disease caused by this novel coronavirus, termed COVID-19, presents a broad clinical spectrum ranging from asymptomatic infection to severe respiratory distress and multiorgan failure, which has caused millions of deaths worldwide [167]. Importantly, SARS-CoV-2 pathogenesis extends beyond severe acute respiratory syndrome, manifesting as a systemic disease involving vascular and hematological components [168].

SARS-CoV-2, the causative agent of COVID-19, is a positive-sense single-stranded RNA virus that shares 79% nucleotide sequence similarity with SARS-CoV [169]. The structure of SARS-CoV-2 is highly characteristic, consisting of nucleocapsid (N), membrane (M), envelope (E), and spike (S) proteins. The spike protein is responsible for binding to its receptor, angiotensin-converting enzyme 2 (ACE2), which is widely expressed on cells of the respiratory tract [170,171]. Once infection is established, viral replication occurs within the respiratory tract, triggering the recruitment of immune cells to the site of infection, production of pro-inflammatory cytokines and chemokines, increased permeability across the pulmonary endothelium, and consequent tissue damage mechanisms that are closely associated with SARS pathogenesis [172,173].

In addition to the pulmonary involvement induced by infection, coagulation abnormalities have emerged as significant contributors to the morbidity and mortality associated with the disease. Hypercoagulable states have been frequently observed in patients infected with SARS-CoV-2. Decreased levels of circulating platelets have been associated with more severe disease outcomes [174]. Furthermore, thrombotic events such as venous and arterial thromboembolism have been correlated with increased mortality among infected individuals [175]. Consistently, microvascular thrombosis was ten times higher in pulmonary histopathological studies from patients who expired from COVID-19 than IAV pneumonia [176]. This section discusses thromboinflammation mechanisms related to SARS-CoV 2 (Figure 2).

Thrombocytopenia can be one of the outcomes of SARS-CoV-2 infection and is associated with disease progression and severity [165]. However, the reduction in platelet count is multifactorial and may involve various mechanisms linked to a complex host–pathogen interaction. Furthermore, SARS-CoV-2 infection triggers an immune response that increases pro-inflammatory cytokine levels, which interfere with CD34+ hematopoietic stem cells, impairing megakaryopoiesis and leading to platelet depletion [167]. Interestingly, SARS-CoV-2 has been detected in megakaryocytes (from both the bone marrow and lungs) and in the platelets from surviving and non-surviving COVID-19 patients, indicating direct infection of these cells [177]. Additionally, transmission electron microscopy analysis revealed the presence of viral particles within bone marrow megakaryocytes from thrombocytopenic and ICU-admitted patients, along with a reduction in these cells, suggesting a direct relationship with the thrombocytopenia observed in clinical findings [178,179].

Regarding the inflammatory response in COVID-19, it is well established that viral infection promotes the recruitment and activation of immune cells, such as macrophages, neutrophils, and dendritic cells, as part of the host defense mechanism. This process triggers the release of several inflammatory mediators, including IL-1β, IL-6, TNF, IFN-γ, IL-12, IL-15, and IL-18 [166]. The heightened inflammatory environment contributes to platelet activation, which may also occur through formation of immune complexes involving IgG binding to the spike protein S1 and subsequent crosslinking of the FcγRII receptor on platelets [180]. Platelet activation is further evidenced by increased P-selectin expression and the formation of platelet–leukocyte aggregates [181,182], mechanisms that enhance thrombus formation and decrease circulating platelet counts. In patients with severe COVID-19 requiring mechanical ventilation, endothelial damage and morphological alterations in pulmonary capillaries have also been reported, leading to platelet activation, aggregation, and increased consumption [183]. A recent study identified the platelet enzyme 12-lipoxygenase (12-LOX) as a protective factor during SARS-CoV-2 infection. The deficiency of 12-LOX was associated with exacerbated inflammation and worse disease outcomes. Expressed in platelets, 12-LOX regulates arachidonic acid metabolism and generates bioactive lipid mediators, including 12-HETE and 12-HETrE, which modulate both platelet activation and inflammatory signaling. In mice lacking 12-LOX, SARS-CoV-2 infection triggered an exacerbated pulmonary response characterized by increased leukocyte infiltration [184]. Additionally, platelets from patients with community-acquired pneumonia, with or without COVID-19, release chemokines, PAI-1, and CD40 ligand. Enhanced release of P-selectin, CD40 ligand, PAI-1, and CCL5 mediated via GPVI was linked to a higher risk of requiring invasive or non-invasive ventilation and/or mortality in COVID-19 patients [185].

Several studies have demonstrated that platelet activation and the formation of platelet–monocyte aggregates are closely associated with disease severity in patients with COVID-19. In severe cases, platelets promote TF expression by monocytes through signaling pathways mediated by P-selectin and integrin αIIb/β3 [186]. Besides inducing TF, P-selectin and integrin-mediated adhesion induces the secretion of inflammatory cytokines and chemokines by the monocyte [187]. Interestingly, monocyte activation was amplified by TF signaling, which induced the secretion of IL-1β and TNF-α in this model [187]. In addition to promoting leukocyte adhesion, activated platelets release mediators that favor thrombosis and inflammation, such as soluble CD40 ligand (sCD40L). Activated platelets also produce lipid mediators such as thromboxane A2 (TXA2) [188] and PAF [179], which are directly associated with vasoconstriction and the formation of platelet aggregates, contributing to a hypercoagulable state, thrombus formation, and heightened inflammation. When bound to endothelial cells, sCD40L induces the expression of adhesion molecules (e.g., ICAM-1, VCAM-1, and E-selectin), which are involved in leukocyte recruitment [189,190]. Recently, a combination of platelet and endothelial cell transcriptomics revealed the S100A8/A9 gene product myeloid-related protein (MRP) 8/14 as a main platelet-derived mediator inducing endothelial thromboinflammation in severe COVID-19 [191].

SARS-CoV-2 infection activates an inflammatory response characterized by the production of several pro-inflammatory mediators that can disrupt crucial pathways, including thrombotic and fibrinolytic processes, as well as those that regulate endothelial tight junctions [192]. This imbalance and inflammatory stimulation promote the recruitment of inflammatory cells to the site of infection, leading to increased endothelial permeability and consequent vascular leakage. It has been demonstrated that neutrophils recruited to the tissue produce NETs in response to infection, which contribute to the formation of platelet–neutrophil aggregates and thrombotic events in COVID-19 patients [193,194,195].

In COVID-19, endothelial dysfunction due to vascular damage or inflammation exposes vWF, promoting platelet activation, adhesion, and aggregation [196]. The inflammatory environment promotes dysregulation between plasminogen activator inhibitor-1 (PAI-1) and the fibrinolytic system, both of which are key regulators of clot formation and dissolution [197]. Fibrinogen is a plasma glycoprotein with a fundamental role in blood coagulation. Upon conversion to fibrin by thrombin, it contributes to clot formation and provides the structural framework for platelet aggregates, supporting platelet adhesion to the vascular endothelium. Under physiological conditions, this process facilitates vascular repair and prevents bleeding [188]. In the context of COVID-19, negative regulation of the fibrinolytic pathway due to elevated PAI-1, the main inhibitor of fibrinolysis, leads to excessive production of fibrinogen, promoting a prothrombotic hypercoagulable state and endothelial dysfunction via thrombus formation. These thrombi can develop in both large vessels and the microvasculature [189].

Hypercoagulability has been associated with increased fibrin deposition in COVID-19 patients [20,190]. During fibrinolysis, when fibrin clots are degraded by plasmin, D-dimer fragments are released. Elevated levels of D-dimers are indicative of hypercoagulability and excessive activation of the coagulation cascade, making them an important biomarker for COVID-19 severity [198,199]. Understanding these interactions is crucial for unraveling the mechanisms behind the severe thrombotic outcomes that affect many patients with COVID-19. Platelet activation and platelet–leukocyte aggregates’ formation significantly contribute to an increased risk of thrombotic events and vascular complications.

Understanding these events is crucial for predicting clinical outcomes, as the symptoms of respiratory viral infections often overlap [200]. Both IAV and SARS-CoV-2 infections upregulate endothelial adhesion molecules, such as VCAM-1 and ICAM-1, which promote platelet–neutrophil aggregation and stimulate the formation of neutrophil extracellular traps (NETs). These cellular interactions enhance the release of pro-inflammatory cytokines, including IL-6 and TNF, amplifying the acute inflammatory response. This excessive inflammation contributes to tissue damage and can ultimately progress to acute respiratory distress syndrome (ARDS).

## 6. The Evidence of Thromboinflammation and Post-Acute Viral Sequelae

Beyond the acute phase of COVID-19, increasing evidence highlights thromboinflammation as a central mechanism in the post-acute sequelae of SARS-CoV-2 infection (long COVID), a condition marked by persistent or newly emerging symptoms after resolution of the acute illness. Several studies report that, months after infection, patients continue to display elevated endothelial activation markers, such as von Willebrand factor, along with fibrinolysis-resistant microclots, indicating sustained hypofibrinolysis and ongoing vascular dysfunction [201,202]. Increased fibrinogen deposition has also been detected in the lungs and brain of COVID-19 patients, correlating with disease severity and serving as a potential biomarker for long COVID [20,203,204]. Furthermore, fibrin can directly bind the SARS-CoV-2 spike protein, generating pro-inflammatory clots that intensify systemic thromboinflammation and contribute to COVID-19-related neuropathology [20].

This prothrombotic state is further reinforced by persistent activation of both the classical and alternative complement pathways, leading to dysregulated formation of the terminal C5b-9 complex, which exacerbates tissue injury and endothelial dysfunction [205]. At the cellular level, patients up to 12 months post-infection display monocyte–platelet aggregates and platelet hyper-reactivity, indicating long-lasting platelet activation [205]. Another key mechanism involves the persistence of SARS-CoV-2 RNA and proteins in various tissues, including the small intestine, even in mild cases often accompanied by reactivation of latent herpesviruses such as Epstein–Barr virus (EBV) and cytomegalovirus (CMV) [206,207]. This phenomenon parallels the behavior of persistent neurotropic viruses and may sustain chronic low-grade inflammation, neuroinflammation, and prolonged clinical symptoms [206,207]. Collectively, the convergence of viral persistence, latent virus reactivation, and ongoing thromboinflammatory activation establishes a plausible pathophysiological axis underlying long COVID.

Similar mechanisms are also observed in DENV infection, in which endothelial dysfunction and direct viral effects contribute to neurological complications. In the central nervous system (CNS), cases of ischemic or hemorrhagic stroke and encephalitis have been reported [208,209]. In the peripheral nervous system, manifestations include long thoracic nerve palsy, abducens nerve palsy, facial palsy, brachial neuritis, myositis, hypokalemic paralysis, and Guillain-Barré syndrome. Ocular complications, including maculopathy, optic neuropathy, and subconjunctival and vitreous hemorrhages, have also been described [210,211].

Encephalopathy is the most frequent neurological manifestation of dengue. Laboratory tests often reveal marked elevations in serum transaminases, hyponatremia, and hypoxia. In Thailand, for example, encephalopathy was reported in nearly half of the children with neurological manifestations associated with DENV infection [212,213,214]. The pathophysiology appears to be multifactorial, involving cerebral edema, hypoxia, and systemic endothelial dysfunction [212,213,214] largely resulting from the thromboinflammatory response triggered by the virus. Another rare but significant complication is ischemic and/or hemorrhagic stroke, reported in patients with confirmed DENV infection [215,216,217,218]. Although the specific mechanisms are not yet fully elucidated, there is evidence that thromboinflammation plays a central role [210]. Studies indicate that interactions among platelets, regulatory T cells, and endothelial cells contribute to thrombus formation, inflammatory activation, and tissue injury, with the CD84 molecule identified as a key mediator at the platelet–T cell interface during ischemic stroke [219].

Clinical manifestations characterized by tremors, rigidity, and bradykinesia have also been reported in patients with DENV infection [220,221]. These cases are classified as viral parkinsonism, a term used to describe syndromes that mimic Parkinson’s disease in association with infectious processes. The mechanism by which DENV triggers these manifestations is not yet fully understood. Evidence suggests that the virus can cross the blood–brain barrier via cytokine-induced increased permeability or through the direct action of viral proteins, such as NS1 [222]. Recent studies have shown that DENV NS1 protein modulates microRNA expression in human cerebral microvascular endothelial cells, compromising endothelial barrier integrity [222]. Moreover, NS1 promotes degradation of the endothelial glycocalyx (EGL), a membrane-associated network of proteoglycans and glycoproteins lining the vascular endothelium, through sialic acid cleavage and the release of heparan sulfate proteoglycans, resulting in increased endothelial permeability [74].

Viral infection-associated parkinsonism is characterized by the presence of at least two of the following symptoms: tremor, bradykinesia, rigidity, and postural instability. Movement disorders related to DENV infection have been widely described in the literature [223]. Furthermore, recent studies indicate that long-term neurological complications associated with viral infections are becoming increasingly frequent, highlighting the need for research aimed at developing therapeutic strategies for these patients.

## 7. Antithrombotic Strategies in Viral Infections: Anticoagulation and Antiplatelet Therapy

The recognition of thromboinflammation as a central component of COVID-19 pathophysiology has spurred extensive research into therapeutic strategies aimed at mitigating the disease’s prothrombotic effects. High-quality randomized clinical trials have demonstrated the efficacy and safety of anticoagulant therapy in hospitalized COVID-19 patients [224,225,226]. Among the agents investigated, heparin is notable due to its widespread use across various regimens and routes of administration. Studies indicate that both low-molecular-weight heparin (LMWH) and unfractionated heparin are associated with improved clinical outcomes. Therapeutic-dose LMWH has been linked to increased survival until hospital discharge, as well as a reduced need for cardiovascular and respiratory support [227]. In addition to systemic administration, alternative approaches such as nebulized heparin have shown efficacy in patients hospitalized with secondary COVID-19 pneumonia [228]. Previous evidence also indicates that inhaled heparin can improve pulmonary coagulopathy and reduce the need for mechanical ventilation ARDS [229]. Its mechanism of action involves inactivating thrombin and factor Xa via interaction with antithrombin, thereby interrupting the coagulation cascade [230]. A recent meta-analysis further reinforces the role of anticoagulation in the context of COVID-19 [231].

Beyond anticoagulant therapies, antiplatelet agents such as aspirin have also been investigated. Experimental and observational studies suggest that aspirin is associated with a reduced incidence of ARDS and improved survival in cases of acute lung injury [232]. As a widely available and low-cost drug, aspirin has been shown to decrease thrombotic events and attenuate in vitro platelet hyperactivity in patients with SARS-CoV-2 infection [233]. Clinical studies have further reported that aspirin use is associated with a lower risk of 28-day in-hospital mortality [234]. However, findings remain somewhat controversial: the RECOVERY trial indicated that, although aspirin did not reduce overall mortality, it was associated with a modest increase in 28-day hospital discharge rates [235].

Meta-analyses indicate that patients with severe COVID-19 have a significantly higher risk of acute cerebrovascular events, heart failure, and myocardial infarction compared to patients with non-severe disease [236]. In this context, P2Y12 receptor antagonists such as clopidogrel, prasugrel, and ticagrelor, widely used in the treatment of ischemic stroke and acute coronary syndrome, have been proposed as potential therapeutic strategies [232,237]. A review conducted by Bikdeli and colleagues (2020) compiled the main drugs with potential roles in modulating COVID-19-associated thromboinflammation, highlighting the importance of investigating antithrombotic agents in this setting [232].

On the other hand, the use of antiplatelet agents in patients with dengue remains controversial due to the increased risk of bleeding associated with thrombocytopenia. A cohort study involving adults with laboratory-confirmed dengue evaluated individuals who were previously receiving aspirin or clopidogrel for cardiovascular diseases [238]. The study compared patients who continued therapy during the thrombocytopenic phase with those who suspended it. The results indicated that therapy’s interruption did not increase the risk of cardiovascular or cerebrovascular events, while continuation of therapy did not worsen the clinical course. Therefore, decisions regarding antiplatelet therapy should be individualized, considering both the bleeding risk and the patient’s underlying cardiovascular condition [238].

In the management of dengue-induced thrombocytopenia, a narrative review suggested the use of thrombopoietin receptor (TPO-R) agonists, such as eltrombopag and romiplostim, the latter approved by the FDA in 2018 for idiopathic thrombocytopenia [239]. These drugs stimulate platelet production by activating thrombopoietin receptors on megakaryocytes and their precursor cells, thereby increasing platelets’ release into circulation. Evidence indicates potential efficacy of these agents in severe cases of thrombocytopenia associated with dengue virus infection [240,241,242,243]. Additionally, transcriptomic and bioinformatic analyses using the Connectivity Map (CMap) have identified potential drug repurposing strategies, including enalapril, traditionally indicated for hypertension, heart failure, and left ventricular dysfunction [244,245]. These agents act as angiotensin-converting enzyme (ACE) inhibitors, reducing angiotensin II and aldosterone levels, thereby lowering peripheral resistance and increasing renin activity. In murine models, enalapril and losartan reduced viral uptake and interleukin-1 production by macrophages during dengue infection, suggesting additional anti-inflammatory and antiviral effects [246].

In the context of influenza-induced pneumonia, a murine study demonstrated that clopidogrel reduced platelet activation and the formation of platelet–leukocyte aggregates [247]. However, this effect alone was insufficient to decrease mortality. Notably, when clopidogrel was combined with oseltamivir, a significant improvement in disease outcomes and survival was observed in the animal model, indicating a synergistic effect between the drugs. These findings suggest that in severe influenza pneumonia, the combination of platelet activation blockade with clopidogrel and viral replication inhibition with oseltamivir may constitute an effective therapeutic strategy [247].

## 8. Conclusions

Thromboinflammation represents a critical interface between the immune and hemostatic systems during viral infections, playing a major role in the pathogenesis and clinical severity of diseases such as dengue, chikungunya, influenza, and COVID-19. A detailed understanding of the molecular and cellular mechanisms driving this dysregulated response is essential for developing more effective therapeutic strategies. Interventions targeting platelet activation, inflammation, and coagulation may provide promising approaches to reduce the morbidity and mortality associated with these infections. Ultimately, advancing our knowledge of virus-induced thromboinflammation could enable more targeted therapies and improve the clinical management of affected patients.

## 9. Methodology

Bibliographic searches for relevant electronic sources were carried out throughout the duration of this study. The Medline/PubMed database (https://pubmed.ncbi.nlm.nih.gov/, accessed on 10 June 2025) and the CAPES Periodicals Portal (Coordination for the Improvement of Higher Education Personnel—CAPES/MEC) (https://www.periodicos.capes.gov.br, accessed on 10 June 2025) were used for data retrieval. Original research articles, review papers, and book chapters in English were selected according to their relevance to the study’s subject matter. The search strategy included the following keywords: thromboinflammation; endothelial dysfunction; thrombocytopenia; viral infection; platelet activation; influenza; chikungunya; SARS-CoV-2; dengue. persistence of platelet activation; neurological sequelae; viral parkinsonism.

**Table 1:** Thromboinflammatory pathways in different viral infections.

Thromboinflammatory Pathways	Dengue	Chikungunya	Influenza	SARS-CoV-2
Platelet activation	Thrombocytopenia [27] Platelet activation with increased P-selectin expression and PF4 release [28,36] Formation of platelet–leukocyte aggregates [59,60]	Platelet activation with increased P-selectin [133]	Platelet activation with increased P-selectin expression [156,157]	Thrombocytopenia [165] Platelet activation with increased P-selectin expression and PF4 release [175,184] Formation of platelet–leukocyte aggregates [186]. Persistent platelet activation [205]
Endothelial dysfunction	Endothelial activation [73,75] Increased vascular permeability [74] Modulation of ICAM-1 and VCAM-1 [29]	Endothelial injury associated with arthritis and chronic inflammation [16]	Endothelial activation Modulation of ICAM-1 and VCAM-1 [151]	Endothelial activation [19] Modulation of ICAM-1 and VCAM-1 [189,190] Increased vascular permeability [189,190]
Abnormalities in coagulation	Increased D-dimer levels [95,96,97] Prolonged activated partial thromboplastin time (aPTT) [95] Prolonged prothrombin time [96] Increased expression of tissue factor in monocytes [106]. Hemorrhage and hypovolemic shock [30]	Increased D-dimer levels [17] Rare cases of deep vein thrombosis [136]	Increased expression of tissue factor [157]	Increased D-dimer levels [198,199] Fibrinogen deposition in tissues [20,203,204] Venous and arterial thrombosis [196] Systemic thromboembolic complications [175]

## Abbreviations

The following abbreviations are used in this manuscript:

aPTT	Activated partial thromboplastin time
ACE2	Angiotensin-converting enzyme 2
ADP	Adenosine diphosphate
ARDS	Respiratory distress syndrome
CHIKV	Chikungunya virus
DENV	Dengue virus
DHF	Dengue hemorrhagic fever
DIC	Disseminated intravascular coagulation
EGL	Endothelial glycocalyx-like layer
GP	Glycoprotein
GPIbα	Glycoprotein Ib alpha chain
HA	Hemagglutinin
HSCs	Hematopoietic stem cells
IAV	Influenza A virus
ICAM-1	Intercellular adhesion molecule-1
ISGs	Interferon-stimulated genes
MIF	Migration inhibitory factors
MCP-1	Monocyte chemoattractant protein –1
MKs	Megakaryocytes
MLR	Monocyte-to-lymphocyte ratio
MMPs	Matrix metalloproteinases
MRP	Myeloid-related protein
NA	Neuraminidase
NAP-2	Neutrophil-activating peptide 2
NETs	Neutrophil extracellular traps
NLR	Neutrophil-to-lymphocyte ratio
NO	Nitric oxide
NS1	Non-structural protein 1
PAF	Platelet-activating factor
PAI-1	Plasminogen activator inhibitor-1
PAR	Protease-activated receptor
PDW	Platelet distribution width
PF-4	Platelet factor 4
PLR	Platelet-to-lymphocyte ratio
PMAs	Platelet–monocyte aggregates
PSGL-1	P-selectin glycoprotein ligand-1
PT	Prothrombin time
ROS	Reactive oxygen species
SARS	Severe acute respiratory syndrome
sCD40L	Soluble CD40 ligand
SD	Severe dengue
TF	Tissue factor
TLR	Toll-like receptor
TXA2	Thromboxane A2
VCAM-1	Vascular cell adhesion molecule-1
VEGF	Vascular endothelial growth factor
VLA-4	Very late antigen-4
